# Refusing to tolerate ongoing prejudicial behavior toward immigrants: Together we can object to prejudicial flag displays

**DOI:** 10.3389/fpsyg.2022.981065

**Published:** 2022-10-12

**Authors:** Nicolay Gausel, Mariëtte Berndsen

**Affiliations:** ^1^Faculty of Social Sciences, University of Stavanger, Stavanger, Norway; ^2^College of Education, Psychology, and Social Work, Flinders University of South Australia, Adelaide, SA, Australia

**Keywords:** social mobilization, racism, shame, nation, unjust, prejudice, national flag

## Abstract

Over recent years, immigrants have been met with unjust prejudiced behavior instead of warm welcome. However, not all citizens of a nation endorse such behavior, instead they try to oppose it through social mobilization. In the context of an ongoing situation where the national flag is used as a prejudiced means to exclude immigrants, individuals who felt attached with all members of the nation felt significantly more shame for the unjust than individuals who glorify their nation. Consequently, attached identifiers expressed a significantly greater motivation than glorified identifiers to start thinking about social mobilization to reclaim the meaning of the flag as a symbol of inclusion, not exclusion. The current study contributes to the growing debate on how immigrants are received, and it helps explain how national identification and feelings such as shame motivate individuals to start thinking about objecting to prejudicial flag displays.

## Introduction

“You must not so easily well tolerate the unjust that does not affect yourself” (Øverland, 1936, translated by us).

To realize ongoing immorality but doing nothing about it seems to be all too easy in this world. On a daily basis, we are fed with news about deep injustice and exploitation of humans on the run from wars, political conflicts, starvation, or economic hardships. Through media we witness how refugees and migrants are being beaten and chased along the borders of Europe, the United States or the shores of Australia, and when they finally make it through, they are met with exclusion and aggressive assaults by flag-waiving nationalist. And it dawns upon us that in this unfair world there is little that can be done to change such immoral, prejudiced behavior.

Or is there?

Realizing there is little one can do on one’s own to change large-scale immorality like prejudice; change might still be achieved through social mobilization of like-minded others for collective efforts. Even though research on shame as a motivator for social mobilization is scarce (but see Shepherd et al., 2013), we believe shame for immorality should be an especially potent motivator to correct ingroup failure as shame is commonly agreed upon as motivating individuals to take action to alleviate the shame they feel (e.g., Tangney and Dearing, 2002; Gausel and Leach, 2011; for a review see Gausel, forthcoming^1^). Hence, in the case of an ongoing, seemingly irreparable failure—as with nationwide prejudiced flag-displays—we believe shameful individuals will be willing to start thinking about social mobilization to collectively object to the ongoing prejudice. Specifically, we anticipate that people who feel ashamed will be those who have an attached identification (i.e., those who are inclusive of and feel attached to all national members regardless of who they are)—in contrast to those who have a glorified identification (i.e., those who idolize and glorify their nation). Following this, we expect those who glorify their nation to be less motivated to think about a call for social mobilization, while those who feel attached to all members of the nation should be more motivated.

## Objecting to ongoing immorality through social mobilization

Where a single individual action is not enough to stop an ongoing injustice, people often mobilize others into collective action to change the status quo (McGeer, 2004; Rogers et al., 2018). In fact, the more people believe their individual action would be ineffective, the more they try to marshal others to gain a collective benefit (Rogers et al., 2018). It is especially under societal instability that these collective actions meant to benefit oneself and others seem to be most powerful (van Zomeren, 2013). For example, it was the instability regarding the termination of the Gaza war in 2008 that predicted collective actions among Jewish Israelis to press on to deliver humanitarian aid to Palestinian citizens (Halperin and Gross, 2011). Thus, social mobilization clearly promotes aims that cannot be accomplished on an individual level.

### National identification

In order to mobilize fellows for collective action one must be able to identify with them. In the current study, we therefore focus on national identification and how people distinguish between themselves and others within in a nation (Reicher and Hopkins, 2001). According to Roccas et al. (2006), identification on a national level can be thought of in two main ways; as glorifying identification and as attached identification.

People who are glorifying identifiers likely require unconditional love for the nation and their symbols, such as the national flag (Roccas et al., 2008). They tend to reject criticism directed toward shortcomings of the nation and perceive themselves as superior compared to other national or ethnic groups (Roccas et al., 2006; Berndsen and Gausel, 2015). Perhaps therefore, glorifying identifiers encourage a monoculture in which social equality between the majority and ethnic minorities is viewed inappropriate and thus, unaccepted (Leidner, 2015). Consequently, they express prejudice toward immigrants and other ethnic minorities (Berndsen and Gausel, 2015, 2019).

In contrast to glorifying identifiers, attached identifiers express an inclusive commitment to all national members; regardless of who they are (Berndsen and McGarty, 2012). Hence, they tend to oppose social inequalities (Berndsen and Gausel, 2019), and if there are wrongs committed within the nation, attached identifiers typically express a critical attitude toward the immoral behaviors committed by fellow nationals (Roccas et al., 2006; Leidner et al., 2010; Berndsen and Gausel, 2015, 2019). Consequently, attached identifiers are willing to mobilize others to stop the ongoing injustice (Berndsen and Gausel, 2015, 2019) and engage in pro-social repair behaviors meant to support harmed group members (Roccas et al., 2006, 2008; Leidner et al., 2010).

### Ingroup immorality and shame

Appraising moral wrong give rise to moral emotions (e.g., Mackie et al., 2000) that promote actions meant to mend the moral defect exposed by the failure and thus repair the consequences for victims (Gausel et al., 2012; Imhoff et al., 2012; Shepherd et al., 2013). One of the key emotions that arise from becoming aware of a moral wrong is the feeling of shame (Gausel and Leach, 2011) Recent research has demonstrated that when shame is felt, it motivates a host of different pro-social reactions aimed to end the immorality and repair consequences of the failure (for reviews, see Gausel and Leach, 2011; Leach and Cidam, 2015; Gausel, forthcoming; see footnote 1).

However, in order to feel moral emotions, such as shame, the self needs to be activated (e.g., Gausel, forthcoming; see footnote 1), either through *personal* actions (Tangney and Dearing, 2002; Gausel et al., 2016) or actions by others whom one identifies with, i.e., *ingroup* members (Schmader and Lickel, 2006; Lickel et al., 2011; Gausel et al., 2012, 2018; Gausel and Brown, 2012). In terms of shame and promoting collective action, Shepherd et al. (2013) found initial support across three studies that anticipated group-based shame predicted a motivation to engage in collective action against ingroup transgression. By such, there is grounds to expect that in the case of an ongoing *ingroup* immorality, members of the ingroup (i.e., Australians) should feel shame when they appraise the ongoing prejudice as immoral and feeling shame should then predict a motivation to start thinking about collective action.

## The current study

In 2005, European Australians waving the Australian flag violently attacked Australians of supposedly Middle-Eastern appearance in the “Cronulla riots,” a suburb in Sydney (for a review, see Berndsen and Gausel, 2019). In the years following the riots, prejudicial use of the Australian flag kept growing in strength (Barton and Beniuk, 2012), and about half of Australians who had national flags on their cars on Australian Day vented prejudiced opinions toward immigrants and asylum seekers (Weber, 2012; see also, Fozdar et al., 2014, showing that hostile flag displays occurred also in Western Australia). The tendency to use flag-displays to express prejudiced attitudes was so widespread that it led to an ultimate ban of Australian logos from Australian Day clothing in two major supermarket chains throughout Australia (Anderson, 2014). It is within this societal context where the national flag has become a symbol for prejudice (and not patriotism or fellowship as it typically means in a context where the flag is not associated with aggression or exclusion of minorities) that we investigated flag-displays. As this is an ongoing, nation-wide prejudice, we anticipate glorifying identifiers to disagree feeling shame for the ongoing prejudice, and thus, be indifferent toward any change of status quo: they simply see no wrong with expressing prejudice toward immigrants and other ethnic minorities. In contrast, we anticipate attached identifiers to agree to feel shame for fellow national’s ongoing prejudice toward immigrants. Realizing there is little they themselves can do to change the status quo; we expect them to be motivated to think about mobilizing others to end the ongoing prejudice.

## Materials and methods

### Participants and design

Participants were 70 students from an Australian university in South Australia. Twelve participants were excluded because of missing data. Of the 58 remaining participants, 78% were female and 22% were male. Their age ranged from 19 to 48 years (*M* = 23.91; *SD* = 7.96). In this sample, 88% had the “Australian” nationality, 7% a “European” nationality, and 5% a “Singaporean” or “Canadian” nationality. All participants had lived more than 5 years in Australia (range 5–54 years). The study involved a between-groups design including a manipulation of national identification (i.e., attached identification vs. glorified identification).^1^

### Stimulus materials and procedure

Participants received a paper and pencil questionnaire that started with the manipulation of national identification. Based on previous research (Roccas et al., 2006; Berndsen and Gausel, 2015), we expected that focusing on discrepancies between the “ideal self” of the nation—as if all was perfect, and the “actual self” of the nation—with all its flaws, should increase an awareness of the nation’s moral inadequacies. This discrepancy between the ideal and the actual self should promote an attached form of identification. Hence, half of participants were allocated to this “*attached identification*” condition. They were asked to think of three attributes of Australians that they would like to find in Australians in order to agree with the sentence: “*I love Australia and viewing myself as an Australian is important to me*.” On the other hand, if participants focus solely on positive attributes of the nation no discrepancies should be awakened. Here, a glorified image of the “nation” will dominate and promote an overly positive view of the nation. Hence, the other half of participants in our study were allocated to this “*glorified identification*” condition. They were asked to think of three attributes of Australians that lead to agreement with the sentence: “*I love Australia and viewing myself as an Australian is important to me*.”

The manipulation was followed by a manipulation check along with an article from The Weekend Australian newspaper (Overington and Warne-Smith, 2005). The article described that the Cronulla riots were triggered by an assault on European Australian surf life savers by Middle-Eastern Australian youths. A week later there was a large and violent protest rally by European Australians and many of them worn Australian flags as capes while violently and verbally assaulting anyone who looked to be of Middle-Eastern appearance. This was followed by subsequent reprisal attacks on European Australians later that evening.

Next, participants read a short summary of the above-mentioned report (Weber, 2012). Finally, participants completed the questionnaire where the minority group was referred to as “Middle-Eastern Australians” and the white majority as “Australians.” These terms are commonly used in Australian social contexts (National Museum Australia, n.d.).

### Dependent measures

All dependent variables were measured with nine-point scales Likert-type anchor points varying from 1 (strongly *disagree*) to 9 (*strongly agree*). *Glorification of the Australian nation* was measured with four items, (partly adapted from Roccas et al., 2006), and were used to check the manipulation of national identification: “Australia is better than other nations to live in,” “Australia is the greatest on earth,” “Other nations can learn a lot of us,” and “Australians think we are pretty good and indeed we are” (Cronbach’s alpha = 0.88). *Shame* was measured with “I feel ashamed to be an Australian when Australians express negative opinions about Middle-Eastern Australians.” *Personal inability to stop prejudicial flag displays* was measured with “I am unable to stop the prejudicial use of the Australian flag.” *Social mobilization* to object to ongoing prejudicial flag displays was measured with “We must reclaim the Australian flag showing that it is NOT used as a symbol of prejudice.”

## Results

### Manipulation check

The manipulation of national identification was successful. Participants in the “glorifying identification” condition agreed significantly more with the glorification statements (*M* = 6.11, *SD* = 1.42) than those in the “attachment identification” condition who disagreed with them (*M* = 4.78, *SD* = 1.66), *t*(56) = 3.26, *p* = 0.002, *d* = 0.88.

### Experimental results

Table 1 displays descriptive statistics and correlations. Consistent with our predictions, participants in the “attachment identification” condition agreed significantly more with feeling shame about the ongoing prejudice (*M* = 7.26, *SD* = 2.34) than did those in the “glorifying identification” condition; which were, as predicted, indifferent to the feeling of shame (*M* = 5.03, *SD* = 2.47), *t*(56) = 3.44, *p* = 0.001, *d* = 0.95.There were no significant difference between participants in the “glorifying identification” (*M* = 4.03, *SD* = 2.28) and “attached identification” (*M* = 4.83, *SD* = 1.83) on personal ability to stop prejudicial flag displays, *t*(56) = 1.40, *p* = 0.16, *d* = 0.39, as both disagreed to be personally able to stop the use of flag-displays. Consistent with predictions, participants in the “attachment identification” condition agreed significantly more with the thought to socially mobilize to object to ongoing prejudicial flag displays (*M* = 7.04, *SD* = 1.82) than did those in the “glorifying identification” condition (*M* = 5.37, *SD* = 2.10), *t*(56) = 3.14, *p* = 0.003, *d* = 0.85.

**Table 1:** Scale inter-correlations and descriptive statistics.

	Variable	1	2	3	4
					
1	Glorification of the Australian nation	−			
2	Shame	−0.40^*^	−		
3	Personally unable stop prejudicial flag displays	−0.38^*^	0.46^**^	−	
4	Social mobilization to object to ongoing prejudicial flag displays	−0.06	0.48^**^	−0.03	−
					
	*M*	5.59	5.91	4.34	6.03
	*SD*	1.64	2.64	2.13	2.14

*N* = 58. Response scale ranged from strongly disagree (1) to strongly agree (9).

* *p* < 0.01;

** *p* < 0.001.

### Structural regression model

**Figure 1:**
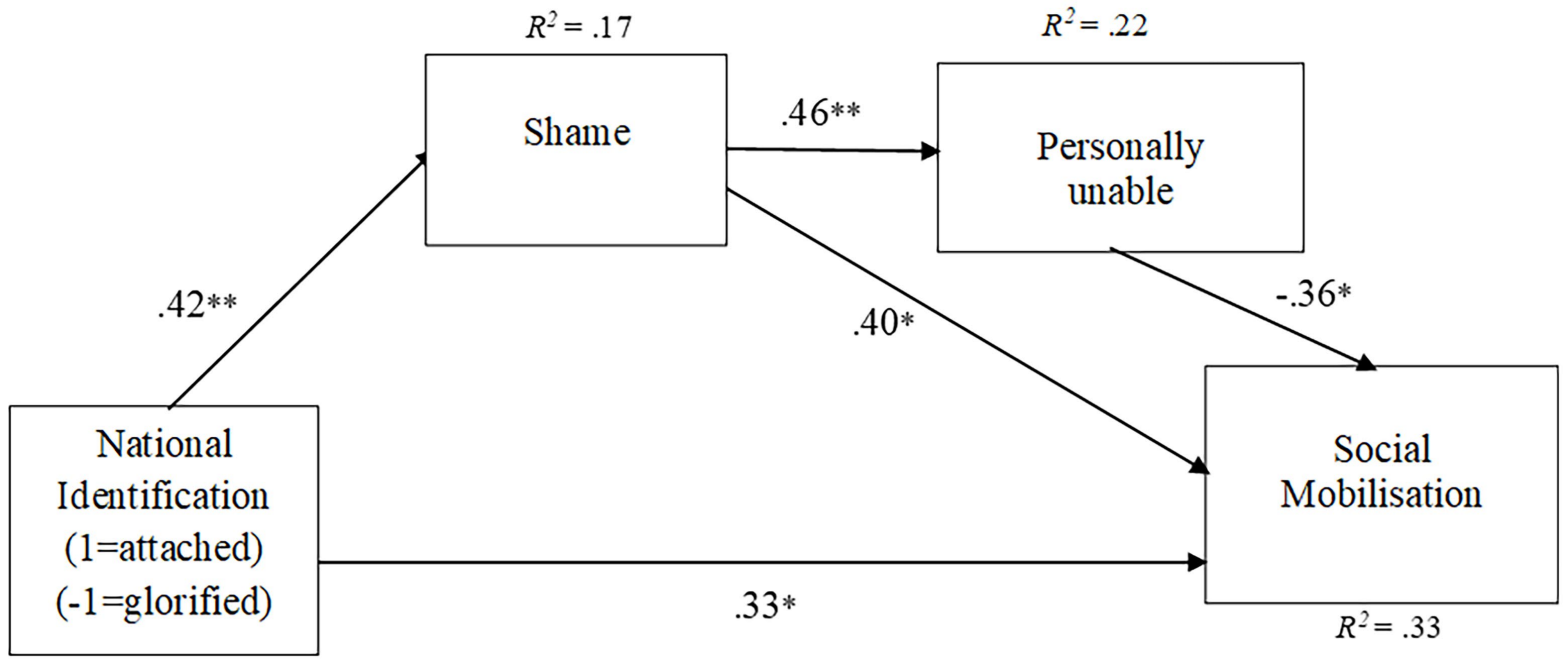
Structural regression model of the relationship between national identification and social mobilization. Solid lines represent statistically significant paths, ^*^p < 0.01 and ^**^p < 0.001.

With the use of AMOS 27, we specified a structural regression model (see Figure 1) coding the two experimental conditions as 1 (“attached identification”) and −1 (“glorified identification”) into a new variable; “national identification.” This allowed us to trace the experimental effect on the dependent variables. Reflecting the experimental results, we allowed “national identification” to directly predict “social mobilization” and indirectly *via* “shame” as well as “personal inability” to stop ongoing prejudicial flag displays. This model had excellent fit [*x^2^*(1) = 0.01, *p* = 0.931, *CFI* = 1.00, *RMSEA* = 0.00]. As seen in Table 2, all indirect effects were significant. Reflecting the experimental results, “attached identifiers” did indeed want to think about “social mobilization” (*β* = 0.33, *p* = 0.006), and they did indeed feel “shame” for the ongoing prejudice (*β* = 0.42, *p* < 0.001). This pattern proved to be the opposite to that of “glorified identifiers,” who did not want to engage in “social mobilization” (*β* = −0.33, *p* = 0.006), and did not feel “shame” for the prejudice (*β* = −0.42, *p* < 0.001). As expected, the more “attached identifiers” agreed to feel shame, the stronger was the realization that one was “personally unable” to stop ongoing prejudicial flag displays (*β* = 0.46, *p* < 0.001). However, and in line with our expectations, the more “shame” felt, the stronger was the motivation to not sit still and watch the injustice unfold, but to start thinking about calling onto others for “social mobilization” to object to ongoing prejudicial flag displays (*β* = 0.40, *p* = 0.002). Unexpectedly, there was a negative link between “personally unable” to stop ongoing prejudicial flag displays and “social mobilization” so that the more “attached identifiers” felt personally unable to end the prejudice, the less they wanted to “socially mobilize” (*β* = −0.36, *p* = 0.004). However, this negative link underlines the potency of “shame” as an emotion that motivates a desire for “social mobilization” that aims at ending prejudice.

**Table 2:** Tests of indirect effects.

IV → mediator → DV	*IE*	*SE*	95% *CI*
National identification → shame → personally unable to stop prejudicial flag displays	0.42	0.16	0.16, 0.78
Shame → personally unable to stop prejudicial flag displays → social mobilization to object to ongoing prejudicial flag displays	−0.11	0.06	−0.25, −0.03
National identification → shame / personally unable to stop prejudicial flag displays → social mobilization to object to ongoing prejudicial flag displays	0.18	0.11	0.01, 0.48

IE, indirect effect; SE, standard error; and 95% CI, 95% confidence interval.

## Discussion

Over recent years prejudice against immigrants has increased. One of the current “trends” in prejudice is using national flags to communicate intolerance and exclusion of immigrants and refugees (e.g., Berndsen and Gausel, 2015). However, such prejudicial flag-displays are not accepted by everyone within a nation. The aim of the current study was therefore to investigate and demonstrate how two different ways to nationally identify would differently impact attitudes toward prejudicial flag-displays.

### A dual national identification

In line with expectations, a glorified view of Australia was endorsed by participants in the “glorifying identification,” while “attached identification” participants disagreed with a glorified view. This successful manipulation of national identification supported earlier research on how national identification can be divided into those who glorify their nation and those who are aware of its flaws and feel attached to all members of the nation (Roccas et al., 2006; Berndsen and Gausel, 2015).

### “Attached identifiers” attitudes toward prejudiced flag-displays

Consistent with predictions, “attached identifiers” agreed with feeling shame in response to ongoing prejudicial flag-displays. This finding supports earlier research of Gausel and Brown (2012) demonstrating how some national members, despite having done nothing wrong personally, still feel shame for other national members prejudiced abuse toward minorities. According to Gausel (forthcoming; see footnote 1), shame felt for other people’s misdeeds is made possible only by the activation of the self of the shameful individual (see also Tangney and Dearing, 2002; Gausel et al., 2016). This means that if someone feels ashamed for other people’s immoral actions, it is because they appraise themselves as being responsible *via* the social identification they share with the wrongdoer (Gausel et al., 2012; Gausel and Brown, 2012). By such, the finding supports a growing body of research demonstrating that people typically feel ashamed about immorality at the hands of others through their shared social identity with the wrongdoers (e.g., Schmader and Lickel, 2006; Brown et al., 2008; Lickel et al., 2011; Gausel et al., 2012, 2018; Berndsen and Gausel, 2015, 2019).

In line with expectations, the more “attached identifiers” agreed to feel shame; the stronger was their realization that they were personally unable to stop the widespread prejudicial flag-displays. This finding supports meta-analysis of Leach and Cidam (2015) demonstrating that shameful individuals will typically withdraw from the shame-eliciting situation if they believe they cannot repair it. However, the result from the current study demonstrates that shameful “attached identifiers” do not always resort to withdrawal and leave it with that, rather, they *approach* the situation and start *thinking* about social mobilization to try to end prejudicial flag-displays. This study therefore lends some initial support for Leach and Cidam (2015) but extends it by supporting Gausel and colleagues’ (Gausel and Leach, 2011; Gausel et al., 2012, 2018; see also Løkkeberg et al., 2021) view of shame as a potent motivator of pro-sociality meant to benefit those harmed by injustice.

Consequently, the more shame felt by “attached identifiers,” the stronger was their motivation toward thinking about social mobilization. This finding supports argument of Rogers et al. (2018) that people try to change things for the better by calling onto like-minded others, and it supports view of van Zomeren (2013) that social mobilization is a path for change—especially under circumstances evident in the Australian context where increased immigration has encouraged civil unrest. Finally, the finding replicates that of Berndsen and Gausel (2015, 2019) who found attached identifiers to be pro-socially objecting to ongoing immorality committed by fellow group-members (see also Roccas et al., 2006, 2008; Leidner et al., 2010).

### “Glorifying identifiers” attitudes toward prejudiced flag-displays

“Glorifying identifiers” did not feel shame for prejudicial flag-displays. In fact, they were indifferent responding to the midpoint of the scale. By such, it seems like the more one identifies in a glorifying way with one’s nation, the less of a problem it is to accept ongoing prejudicial flag-displays meant to exclude immigrants and other ethnic minorities. As shame is a moral emotion (Tangney and Dearing, 2002) that arise from appraising a self-related moral failure (Gausel and Leach, 2011), the lack of felt shame convey the message that “glorified identifiers” do not appraise prejudiced flag-displays as violating a moral norm. This finding is somewhat worrying, as the study demonstrates that merely thinking of positive aspects of ones’ nation seems to bar the capacity to feel a key moral emotion arising from moral failure.

Consequently, as no shame is felt (i.e., no moral norm has been violated), “glorified identifiers” are indifferent toward social mobilization of others. In fact, the structural path model demonstrates that “glorified identifiers” approve *less* on social mobilization through their lack of feeling shame for the prejudice. These results reflect earlier findings by Berndsen and Gausel (2015, 2019) that majority individuals glorifying their nation seem to tolerate prejudice aimed at minorities.

### Possible limitations

There are at least three possible limitations with the study. First, a sample of students may differ from a sample of the wider community. Although this might be true, we find it concerning that students, assumed to be more reflective and liberal, seems indifferent to the ongoing prejudice and feel no shame for it when allocated to an arbitrary “glorified identifiers” condition. Moreover, it is worth noting how media polls in Australia keep finding the population to be split in different attitudes toward the national flag in light of the ongoing prejudice (e.g., Marszalek, 2013). Thus, we see a sample of students to be aligned with the general community. The second possible limitation is the somewhat modest sample size. While we acknowledge this, a power analysis using a *post hoc* G*power analysis (Faul et al., 2007) demonstrated that a sample size of 58 is *sufficient* to reach a medium effect size of *f^2^* = 0.15, an alpha level of 0.05, and 1 predictor (shame) produced a power level of 0.83. Some might say a third limitation is the use of single item measures. They might reason that a single item measurement might be understood differently depending on who you ask. While we acknowledge the ideal would be to measure an attitude (or emotion-experience) through a more complex semantic, integrated cognition-emotion process (Gausel, forthcoming; see footnote 1), one should remember that a single item measurement using the keyword within its meaning-providing context (that is; a full sentence) is more than sufficient to provide the word with semantic meaning (Davidson, 1967) minimizing the risk of semantic misunderstandings (Gausel, 2014).

### Conclusion: A step toward better understanding of ongoing prejudice

With a lower sample size, we cannot draw conclusions in terms of generalization, but we can view our study as a step toward a better understanding of the dynamics between national identification and how to handle ongoing prejudice toward immigrants and refugees. The study demonstrated that those who approve of prejudiced flag-use toward immigrants and refugees are those who identify in a glorifying way with their nation, while those who are motivated to oppose prejudiced behavior are those who identify in an attached way with all members of the nation. We found both national identifying groups to be personally unable to stop prejudicial flag displays, but only attached identifiers agreed to start thinking about collective actions to end ongoing injustice believed to be irreparable for a single individual *via* their agreed shame for the prejudice. In conclusion, the results of the current study can be used to alter the poetic words by Øverland (1936) to conclude that “attached identifiers” do not “*so easily well tolerate the unjust that does not affect yourself*” while “glorified identifiers” can “*easily well tolerate the unjust that does not affect yourself*.”

## Data availability statement

The raw data supporting the conclusions of this article will be made available by the authors, without undue reservation.

## Ethics statement

The studies involving human participants were reviewed and approved by Human Research Ethics, Flinders University. The patients/participants provided their written informed consent to participate in this study.

## Author contributions

NG and MB have contributed equally to the article, but MB designed and collected the data. All authors contributed to the article and approved the submitted version.

## Conflict of interest

The authors declare that the research was conducted in the absence of any commercial or financial relationships that could be construed as a potential conflict of interest.

## Publisher’s note

All claims expressed in this article are solely those of the authors and do not necessarily represent those of their affiliated organizations, or those of the publisher, the editors and the reviewers. Any product that may be evaluated in this article, or claim that may be made by its manufacturer, is not guaranteed or endorsed by the publisher.

## Footnotes

^1^Gausel, (forthcoming). "What is shame? Shame as a relational network of emotion-experience," in Handbook on the Psychology of Morality. eds. N. Ellemers, S. Pagliaro and F. van. Nunspeet (Routledge).

^2^We included more dependent variables in the study than we reported here. A full version of the measures can be provided by the corresponding author.
